# Butyrate Protects Barrier Integrity and Suppresses Immune Activation in a Caco-2/PBMC Co-Culture Model While HDAC Inhibition Mimics Butyrate in Restoring Cytokine-Induced Barrier Disruption

**DOI:** 10.3390/nu15122760

**Published:** 2023-06-15

**Authors:** Sandra G. P. J. Korsten, Herman Vromans, Johan Garssen, Linette E. M. Willemsen

**Affiliations:** 1 Division of Pharmacology, Utrecht Institute for Pharmaceutical Sciences, Utrecht University, 3584 CG Utrecht, The Netherlands; 2 Tiofarma B.V., 3261 ME Oud-Beijerland, The Netherlands; 3 Division of Pharmaceutics, Utrecht Institute for Pharmaceutical Sciences, Utrecht University, 3584 CG Utrecht, The Netherlands; 4 Danone/Nutricia Research B.V., 3584 CT Utrecht, The Netherlands

**Keywords:** butyrate, short chain fatty acids, intestinal epithelial cells, PBMCs, mucosal immunity, in vitro models

## Abstract

Low-grade inflammation and barrier disruption are increasingly acknowledged for their association with non-communicable diseases (NCDs). Short chain fatty acids (SCFAs), especially butyrate, could be a potential treatment because of their combined anti-inflammatory and barrier- protective capacities, but more insight into their mechanism of action is needed. In the present study, non-activated, lipopolysaccharide-activated and αCD3/CD28-activated peripheral blood mononuclear cells (PBMCs) with and without intestinal epithelial cells (IEC) Caco-2 were used to study the effect of butyrate on barrier function, cytokine release and immune cell phenotype. A Caco-2 model was used to compare the capacities of butyrate, propionate and acetate and study their mechanism of action, while investigating the contribution of lipoxygenase (LOX), cyclooxygenase (COX) and histone deacetylase (HDAC) inhibition. Butyrate protected against inflammatory-induced barrier disruption while modulating inflammatory cytokine release by activated PBMCs (interleukin-1 beta↑, tumor necrosis factor alpha↓, interleukin-17a↓, interferon gamma↓, interleukin-10↓) and immune cell phenotype (regulatory T-cells↓, T helper 17 cells↓, T helper 1 cells↓) in the PBMC/Caco-2 co-culture model. Similar suppression of immune activation was shown in absence of IEC. Butyrate, propionate and acetate reduced inflammatory cytokine-induced IEC activation and, in particular, butyrate was capable of fully protecting against cytokine-induced epithelial permeability for a prolonged period. Different HDAC inhibitors could mimic this barrier-protective effect, showing HDAC might be involved in the mechanism of action of butyrate, whereas LOX and COX did not show involvement. These results show the importance of sufficient butyrate levels to maintain intestinal homeostasis.

## 1. Introduction

Non-communicable diseases (NCDs) are a leading cause of mortality, responsible for approximately 74% of all deaths worldwide [1]. NCDs are chronic metabolic or immune disorders, including diabetes, inflammatory diseases, cardiovascular diseases and chronic respiratory diseases, that are not caused by infectious agents. They are characterized by low-grade inflammation, systemically and locally in the gut, and it is hypothesized that these diseases are associated with dysbiosis of the microbiome and a disrupted intestinal barrier [2,3,4,5,6,7,8,9]. Dysbiosis of the microbiome leads to increased influx of macromolecules through the intestinal barrier, such as lipopolysaccharides (LPS), leading to local inflammation in the gut and low-grade inflammation systemically [10,11]. Furthermore, inflammation may establish a positive feedback loop by maintaining the disrupted intestinal barrier, leading to an ongoing influx of macromolecules and consequently resulting in chronic inflammation and the development of NCDs [12,13,14,15,16,17].

The microbiome produces biologically active molecules such as short chain fatty acids (SCFAs) by fermentation of fibers, which may be hampered in NCDs due to dysbiosis [18,19,20,21,22]. SCFAs, especially butyrate, are known for their beneficial effects on systemic inflammation and gut health, including anti-inflammatory and barrier-improving effects. Therefore, SCFAs could be a possible treatment option for patients with NCDs by restoring intestinal homeostasis and intestinal barrier function [23,24,25]. SCFAs have been extensively studied in various in vitro models with intestinal epithelial cells (IEC) or in immune assays using peripheral blood mononuclear cells (PBMCs) revealing the diverse effects of SCFAs. However, these effects have not been studied in Caco-2/PBMC co-culture models allowing the cross talk between IEC and immune cells mimicking their proximity at the mucosal surface while studying immune-mediated barrier disruption.

In IEC models, in particular, butyrate altered the assembly and expression of tight junction proteins and was able to enhance barrier function [26,27,28,29]. Additionally, butyrate reduced the activation status of IEC, thus contributing to homeostasis, by lowering the expression of pro-inflammatory cytokines and chemokines, including Interleukin (IL)-8 and C-X-C motif chemokine ligand 10 (CXCL10) [30,31,32,33]. Butyrate is known as a histone deacetylase (HDAC) inhibitor, which was found to contribute to its anti-inflammatory and barrier-protective effect [34]. Butyrate-induced activation of lipoxygenase was also found to contribute to barrier protection [26].

In PBMC models, butyrate inhibited the production of pro-inflammatory cytokines such as tumor necrosis factor-alpha (TNF-α), interferon-gamma (IFN-γ), IL-1β and IL-6, while increasing the production of anti-inflammatory cytokines such as IL-10 [34,35,36,37,38,39,40]. Moreover, butyrate could modulate the differentiation of T-cells within the PBMC models into different subtypes, such as the promotion of regulatory T-cells [35].

The current study aimed to investigate the effect of butyrate using a Caco-2/activated PBMC co-culture model which makes it possible to study the cross talk between IEC and effector immune responses. This type of research can help to provide a more complete understanding of the complex interactions between these different cell types and how they are affected by butyrate. In this transwell co-culture model, IEC were grown on inserts and PBMCs were added to the basolateral compartment and LPS or αCD3/CD28 were used to activate the PBMCs in the presence or absence of butyrate. Next, the protective effect of butyrate, or the other SCFAs, propionate and acetate, in cytokine-mediated barrier disruption, and the potential involvement of LOX, COX or HDAC inhibition in their mechanism of action was investigated.

## 2. Materials and Methods

### 2.1. Cell Culture

#### 2.1.1. Intestinal Epithelial Cell Culture

Human colorectal adenocarcinoma Caco-2 cells (ATTC, HTB-38, Manassas, VA, USA; passage 36–45) were used as intestinal epithelial cells (IEC) and were maintained in 75 cm^2^ culture flasks (Greiner Bio-One, Kremsmünster, Austria) using culture medium. Culture medium consisted of high glucose Dulbecco’s Modified Eagle Medium (Gibco, Invitrogen, Carlsbad, CA, USA) supplemented with 10% heat-inactivated fetal calf serum (FCS) (Sigma-Aldrich, St. Louis, MO, USA), 1% penicillin/streptomycin (100 U/mL and 100 μg/mL, respectively) (Sigma-Aldrich), 1% non-essential amino acids (Gibco) and 2 mM L-Glutamine (Gibco). Medium was refreshed every 2–3 days and cells were passaged when a confluency of 70–90% was reached. Cells were kept at 37 °C and 5% CO_2_ in an incubator.

#### 2.1.2. PBMC Isolation

Healthy donor buffy coats (Blood Bank, Amsterdam, The Netherlands) were used to isolate human PBMCs by density gradient centrifugation using pre-filled Leucosep™ tubes (Greiner Bio-One). Tubes were centrifuged for 13 min at 1000× *g* after which the PBMC fraction was isolated and washed with phosphate buffered saline (PBS) (Lonza, Basel, Switzerland) supplemented with 2% heat-inactivated FCS and the remaining erythrocytes were lysed using a lysis buffer (4.14 g NH_4_Cl, 0.5 g KHCO_3_, 18.6 mg Na_2_EDTA in 500 mL demineralized water, sterile filtered, pH = 7.4) for 5 min on ice. Finally, the PBMCs were resuspended in RPMI 1640 (Gibco) supplemented with 10% heat-inactivated FCS and 1% penicillin/streptomycin (100 U/mL and 100 μg/mL, respectively). All blood donors, who can volunteer without any selection on age, ethnicity or sex, gave informed consent for the use of their blood for scientific research purposes. In addition, strict conditions for use were set, for which permission was obtained by our research group. 

### 2.2. In Vitro Models

#### 2.2.1. Co-Culture Model and PBMC Stimulation

The Caco-2 cells were seeded in a 12-transwell system with a pore size of 0.4 µm (Costar, Corning Incorporated, Corning, NY, USA) and grown for 3 weeks until they were differentiated to small intestinal epithelial cells before the start of the experiment, as described elsewhere [41]. For the co-culture experiment, the culture medium was changed to RPMI 1640 supplemented with 2.5% heat-inactivated FCS and 1% penicillin/streptomycin (100 U/mL and 100 μg/mL, respectively). In the basolateral compartment, PBMCs at a concentration of 2 × 10^6^ cells/mL were seeded non-activated or activated with 1 μg/mL LPS or a combination of αCD3 and αCD28 (clone CLB-T3/2 and clone CLB-CD28, both 1:10,000, Sanquin, Amsterdam, The Netherlands). In the apical compartment, sodium butyrate (Sigma-Aldrich) was added in a concentration of 0.5, 2 or 8 mM. At t = 24 h, complete apical medium was replaced with fresh culture medium with or without butyrate and 0.5 mL of the basolateral medium was replaced with fresh culture medium without any additions. At t = 48 h, the experiment was ended and the basolateral medium was stored at −20 °C until further analysis.

**Figure 1:**
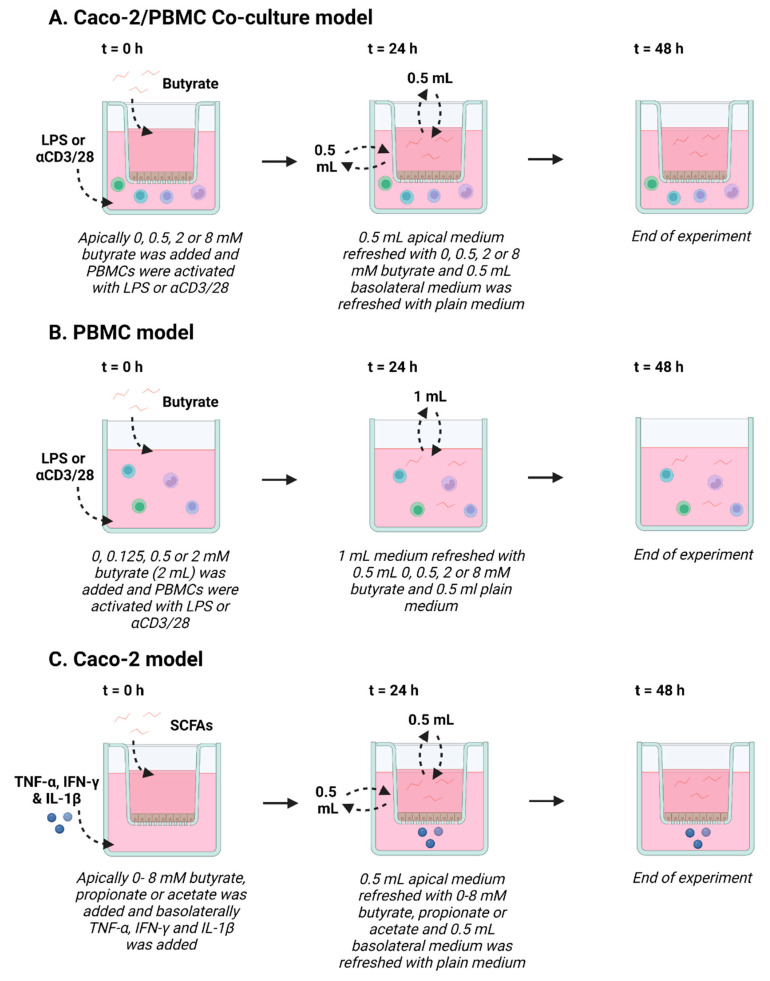
Schematic overview of Caco-2/peripheral blood mononuclear cells (PBMCs) co-culture model (**A**), PBMC model (**B**) and Caco-2 model (**C**). (**A**) In the Caco-2/PBMC co-culture model, 0–8 mM butyrate was added to the apical side of the Caco-2 cells and PBMCs were activated with either lipopolysaccharide (LPS) or αCD3/CD28. After 24 h, apical medium was refreshed with fresh medium containing 0–8 mM butyrate and 0.5 mL of the basolateral medium was refreshed with medium without butyrate. At 48 h, the experiment was ended. (**B**) In the PBMC model, 0–2 mM butyrate was added to the PBMCs, which were activated with either LPS or αCD3/CD28. After 24 h, 1 mL of medium was refreshed with 0.5 mL medium containing 0–8 mM butyrate and 0.5 mL medium without butyrate, to mimic the co-culture model. At 48 h, the experiment was ended. (**C**) In the Caco-2 model, 0–8 mM butyrate, propionate or acetate was added to the apical side of the Caco-2 cells and TNF-α, IFN-γ and IL-1β was added to the basolateral compartment to activate the cells. After 24 h, apical medium was refreshed with fresh medium containing 0–8 mM butyrate, propionate or acetate and 0.5 mL of the basolateral medium was refreshed with medium without short chain fatty acids (SCFAs). At 48 h, the experiment was ended. Created with BioRender.com.

In addition, PBMCs were incubated with butyrate in the absence of Caco-2 cells at a concentration of 0.125, 0.5, 2 mM butyrate in a 12-well plate. At t = 24 h, 1 mL medium was replaced with 0.5 mL fresh culture medium and 0.5 mL fresh culture medium with or without 0.5, 2 or 8 mM butyrate, to simulate the co-culture experiment. At t = 48 h, the experiment was ended and medium was stored at −20 °C up until further analysis. During the experiment, the cells were kept at 37 °C and 5% CO_2_ in an incubator. See Figure 1A,B for an overview of the co-culture and PBMC model.

#### 2.2.2. IEC Model

The Caco-2 cells were seeded in a 12-transwell system with a pore size of 0.4 µm and grown for 3 weeks until they were differentiated to small intestinal epithelial cells before the start of the experiment, as described elsewhere [41]. At t = 0 h, the SCFAs, sodium butyrate (Sigma-Aldrich), sodium propionate (Sigma-Aldrich) and sodium acetate (Emsure, MI, USA) were added to the apical side of the cells and a mixture of pro-inflammatory cytokines, 10 ng/mL IL-1β (Preprotech, London, UK), 100 U/mL IFN-γ (Preprotech) and 10 ng/mL TNF-α (Sigma-Aldrich) was added to the basolateral side of the cells. The SCFAs were dissolved and diluted to the working concentration in culture medium. Butyrate was used at a concentration of 0.5, 1, 2, 4 and 8 mM, propionate was used at a concentration of 2 and 4 mM and acetate was used at a concentration of 4 and 8 mM. The pro-inflammatory cytokines were dissolved in PBS and diluted to the working concentration in culture medium. At t = 24 h, complete apical medium was replaced with fresh culture medium with or without SCFAs and 0.5 mL of the basolateral medium was replaced with fresh culture medium without any additions. At t = 48 h, the experiment was ended and cells were stored in RIPA lysis buffer (Thermo Fisher Scientific, Waltham, MA, USA) at −20 °C until further analysis, even as basolateral medium. During the experiment, the cells were kept at 37 °C and 5% CO_2_ in an incubator. See Figure 1C for an overview of the IEC model.

To investigate the mechanism of action of the short chain fatty acids (SCFAs), LOX inhibitor nordihydroguaiaretic acid (10 μM) or COX inhibitor indometacine (1 μM) (Sigma-Aldrich) were added to the experiment described above. In addition, HDAC inhibitors trichostatin A (1 μM), tacedinaline (2.5, 25, 250 μM), tinostamustine (0.1, 1, 10 μM) and TMP269 (0.1, 1, 10 μM) (MedChemExpress, Sollentuna, Sweden) were added as a treatment instead of the SCFAs to see whether these inhibitors could mimic the effect of the SCFAs.

### 2.3. Barrier Assessment

Barrier integrity of the Caco-2 monolayer was assessed with two different methods, transepithelial electrical resistance (TEER) and a 4 kDa fluorescein isothiocyanate-dextran (FD4) permeability assay.

#### 2.3.1. TEER

The TEER of the monolayer was measured using a Millicell ERS-2 Volt-ohm meter (Merck Millipore, Burlington, MA, USA) at t = 0 h, t = 24 h and t = 48 h. Data were shown as a percentage compared to the TEER of the monolayer at t = 0 h.

#### 2.3.2. 4 kDa FITC-Dextran Permeability Assay

At t = 48 h, a FD4 (Sigma-Aldrich) permeability assay was performed to assess paracellular permeability of the cell monolayer. First, the phenol red-containing medium was discarded, the cells were washed with PBS and the same culture medium without phenol red (Gibco) was added. Before the start of the assay, the cells were left in the incubator for 1 h to become stable again. A concentration of 1.6 mg/mL FD4 was added to the apical side of the IEC and 100 uL samples of the basolateral medium were taken 1, 4 or 24 h after the addition of the FD4 and collected in a white 96-well plate (Corning). The taken samples were measured at Ex/Em = 492/518 nm with Fluorskan Ascent FL (Thermo Labsystems, Waltham, MA, USA).

### 2.4. Viability Assay

After the FD4 permeability assay, the cells were washed again with PBS and the viability of the cells was assessed using the cell proliferation Reagent WST-1 (Roche Diagnostics GmbH, Mannheim, Germany) according to the manufacturer’s instructions. Data were shown as a percentage compared to the control cells.

### 2.5. Enzyme-Linked Immunosorbent Assay (ELISA)

Basolateral supernatant was used to measure the IL-8, IL-17a, IFN-γ, IL-10, TNF-α, IL-1β and IL-6 concentrations present in the medium as a measure for epithelial or immune cell activation. Cytokine and chemokine concentrations were measured using commercially available ELISA kits (Thermo Fischer scientific, Waltham, MA, USA). ELISA was performed according to the manufacturer’s instructions. In short, high-binding 96-well plates (Corning) were coated with capture antibody and incubated overnight at 4 °C; all of the following steps were performed at room temperature. Non-specific binding was blocked for 1 h. After washing, the samples or the standard were added for 2 h. Then, plates were washed and incubated with streptavidin–horseradish peroxidase for 1 h. Subsequently, the plates were washed and incubated in the dark with substrate solution. The reaction was stopped with 1 M H_2_SO_4_ and absorbance was measured at 450 nm in a microplate reader (iMark, Bio-Rad Laboratories, Hercules, CA, USA or GloMax Discover, Promega Corporation, Madison, WI, USA).

### 2.6. Flow Cytometry

At the end of the cell experiments, PBMCs were transferred to a 96-well plate with a U bottom (Corning) for flow cytometry. First, cells were blocked with PBS supplemented with 5% heat-inactivated FCS to prevent unspecific binding, dyed with Fixable Viability Dye 780-APC Cyanine 7 (1:2000; eBioscience, Thermo Fisher Scientific) for 5 min in the dark and washed with FACS buffer (PBS + 2.5% heat-inactivated FCS). Second, cells were incubated for 30 min on ice with titrated volumes of the following antibodies: CD4-PerCP Cy 5.5, CD69-PE, CD25-FITC, CD127-PE Cy 7, CRTH2-APC (all eBioscience), CXCR3-FITC (BD Bioscience, San Jose, CA, USA). After incubation, cells were washed again using FACS buffer and fixed to store them overnight at 4 °C. Cells that were only stained with extracellular antibodies were fixed with IC fixation buffer 1:4 in PBS and cells that were stained extracellularly and intracellularly were fixated and permeabilized with Fix/Perm buffer (Thermo Fisher Scientific). The next day panels with only extracellular staining were washed, resuspended in FACS buffer and measured using a BD FACS Canto II (Becton Dickinson, Franklin Lakes, NJ, USA). Panels with extracellular and intracellular staining were washed and incubated for 30 min on ice with titrated volumes of the following antibodies: FoxP3-eFluor660, RORγ-PE, Tbet-eFluor660 and GATA3-PE (all eBioscience). After incubation, cells were washed, resuspended in FACS buffer and measured using a BD FACS Canto II. Acquired data were analyzed using FlowLogic software (Inivai Technologies, Mentone, Australia).

### 2.7. Western Blot

At t = 48 h, the cells were collected in RIPA lyses buffer supplemented with protease inhibitors (Roche). First, protein concentration was determined using a BCA protein assay kit (Thermo Fisher Scientific) and protein concentrations were equalized across all samples. Bromophenol blue and 2-mercaptoethanol were added to the samples to denature the proteins. Protein samples were then added to a Criterion^TM^ 4–20% Tris-HCl gel (Bio-Rad, Veenendaal, The Netherlands) for separation with electrophoresis. Thereafter, the proteins were transferred from the gel to a polyvinylidene difluoride membrane (Transblot Turbo, Bio-Rad). The membrane was blocked using 5% milk protein (Nutricia protifar, Danone, Hoofddorp, The Netherlands) in PBS containing 0.05% Tween-20. After blocking, the membrane was incubated with primary antibodies overnight at 4 °C. As primary antibodies, zonula occludens-1 (ZO-1) (1:1000, Thermo Fisher Scientific), occludin (1:1000, Thermo Fisher Scientific) (1:1000) and β-actin (1:1000, cell signaling) were used. After incubation, the membranes were washed and incubated with horseradish peroxidase conjugated secondary antibodies (Dako, Santa Clara, CA, USA) for 2 h. Membranes were again washed in PBS containing 0.05% Tween-20 and the proteins on the membranes were visualized using ECL reagent (Bio-Rad). Data were analyzed using Image J version 1.52a.

### 2.8. Statistical Analysis

Results are presented as means (±SEM) as the experiments were performed as at least 4 individual experiments. Statistical analysis was performed using GraphPad Prism 8.4.3 (GraphPad Software, San Diego, CA, USA). The statistical significance of normally distributed data was assessed with the repeated measures one-way ANOVA analysis, followed by Bonferroni’s post hoc test with selected pairs. Statistical significance of not normally distributed data was assessed with the Friedman test with selected pairs, followed by Dunn’s multiple comparisons test. Data presented in the figures are, in some cases, divided into different subsets for statistical analysis. The different subsets are divided by dotted lines. Within these subsets, selected pairs were used for statistical analysis, in which the activated or non-activated condition without butyrate, propionate or acetate was compared with the different concentrations of butyrate, propionate or acetate within the same subset. A paired Student’s *t*-test (for normally distributed data) or Wilcoxon matched pairs signed rank test (for not normally distributed data) was used to compare positive and negative controls, namely, non-activated cells with LPS, αCD3/CD28 or cytokine mix-activated cells. Results were considered statistically significant when *p* < 0.05. Significant differences are shown in the figures as * *p* < 0.05, ** *p* < 0.01, *** *p* < 0.001 and **** *p* < 0.0001.

## 3. Results

### 3.1. Butyrate Improves Intestinal Barrier Function in a Caco-2/PBMC Co-Culture Model

**Figure 2:**
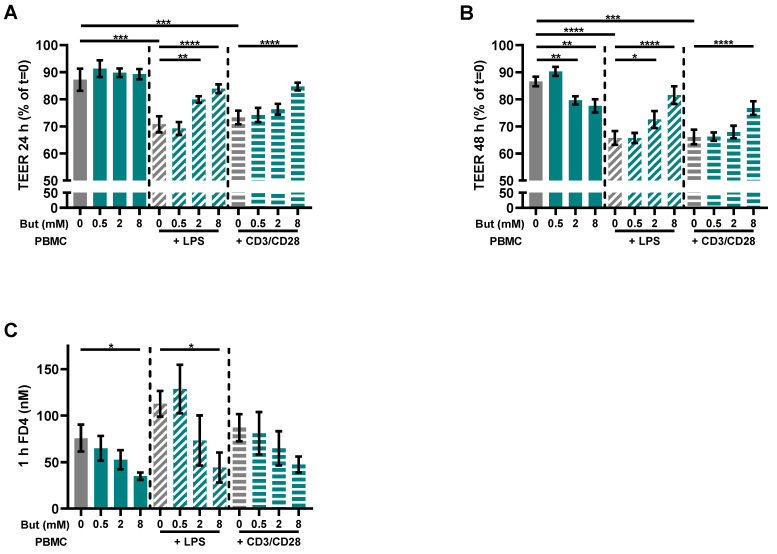
Butyrate protects against barrier disruption in a Caco-2/PBMC co-culture model with LPS- and αCD3/CD28-activated PBMCs. Cells were treated with 0–8 mM butyrate. (**A**) Transepithelial electrical resistance (TEER) values at t = 24 h expressed as percentage of the initial TEER at t = 0. (**B**) TEER values at t = 48 h expressed as percentage of the initial TEER at t = 0. (**C**) 4 kDa fluorescein isothiocyanate dextran (FD4) permeability assay performed at the end of the experiment. Results show the FD4 concentration in the basolateral compartment after 1 h of incubation. Data are presented as mean ± SEM and the datasets used for statistical analysis are divided by dotted lines (N = 6 individual experiments). Significant differences are shown as * *p* < 0.05, ** *p* < 0.01, *** *p* < 0.001, **** *p* < 0.0001 compared to control. But: butyrate; PBMC: peripheral blood mononuclear cells.

To investigate the potential of butyrate to improve the intestinal barrier function, a co-culture model of intestinal epithelial cells (IEC) combined with activated immune cells simulating an effector immune response was used. The PBMCs were activated with either LPS or a combination of αCD3 and αCD28 to provoke a cytokine response contributing to barrier disruption. Both LPS and αCD3/CD28 activation significantly reduced barrier function, as TEER was reduced by approximately 20% (Figure 2A,B). Butyrate protected against inflammatory-induced barrier disruption (TEER) in the activated models. In the LPS-activated model, butyrate protected at a concentration of 2 and 8 mM and in the αCD3/CD28 activated model at a concentration of 8 mM at 24 h and 48 h (Figure 2A,B). In the non-activated model, 2 and 8 mM butyrate slightly reduced TEER values at t = 48 h (Figure 2B). The functional FD4 paracellular permeability in the activated models was not significantly increased as compared to controls (IEC/non-activated PBMCs), but butyrate (8 mM) lowered the basal permeability (Figure 2C). Furthermore, 8 mM butyrate also reduced permeability in the LPS-activated model and the αCD3/CD28-activated model showed a similar pattern (Figure 2C).

### 3.2. Butyrate Modulates Pro-Inflammatory and Regulatory Cytokines Release

**Figure 3:**
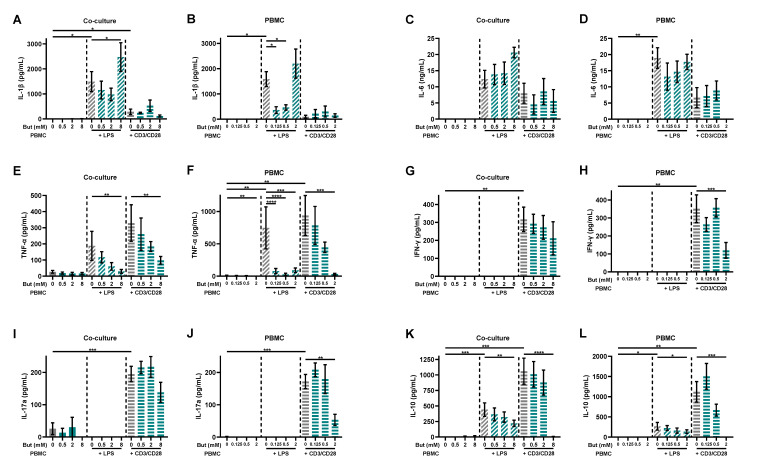
Butyrate modulates cytokine release in a Caco-2/PBMC co-culture model (**A**,**C**,**E**,**G**,**I**,**K**) and PBMC model (**B**,**D**,**F**,**H**,**J**,**L**). PBMCs were activated with LPS or αCD3/CD28 while cells were treated with 0–8 mM butyrate. IL-1β (**A**,**B**), IL-6 (**C**,**D**), TNF-α (**E**,**F**), IFN-γ (**G**,**H**), IL-17a (**I**,**J**) and IL-10 (**K**,**L**) were measured in the supernatant. Data are presented as mean ± SEM and the datasets used for statistical analysis are divided by dotted lines (N = 5–6 individual experiments). Significant differences are shown as * *p* < 0.05, ** *p* < 0.01, *** *p* < 0.001, **** *p* < 0.0001 compared to control. But: butyrate; PBMC: peripheral blood mononuclear cells.

In order to investigate the effect of butyrate on immune activation, various cytokines were measured in the basolateral supernatant of the co-culture model and in the supernatant of the PBMC model. Similar patterns were observed in the results of both models. LPS induced the release of IL-1β, IL-6, TNF-α and IL-10, but not of IL-17A and IFN-γ in both models, although, in some cases, not significantly in one of both models. The induced release of TNF-α and IL-10 was prevented by 8 mM butyrate in the co-culture model and 2 mM butyrate in the PBMC model (Figure 3). Butyrate did not influence the release of IL-6 in both models, while low concentrations of butyrate (0.125 and 0.5 mM) reduced the release of IL-1β in the PBMC model, but not in the co-culture model. However, 8 mM butyrate further enhanced the release of IL-1β in the co-culture model (Figure 3A). αCD3/CD28 induced the release of IL-1β, TNF-α, IFN-γ, IL-17A and IL-10, but not of IL-6 and IL-1β in both models, although, in some cases, not significantly in one of both models. Moreover, in this model, the induced release of TNF-α and IL-10 was prevented by 8 mM butyrate in the co-culture model and 2 mM butyrate in the PBMC model, similar to the LPS-activated models. Butyrate did not influence the release of Il-1β in both models, while 2 mM butyrate reduced the release of IFN-γ and IL-17A in the PBMC model, but had no statistically significant effect in the co-culture model (Figure 3).

### 3.3. Butyrate Modulates T-Cell Phenotypes

**Figure 4:**
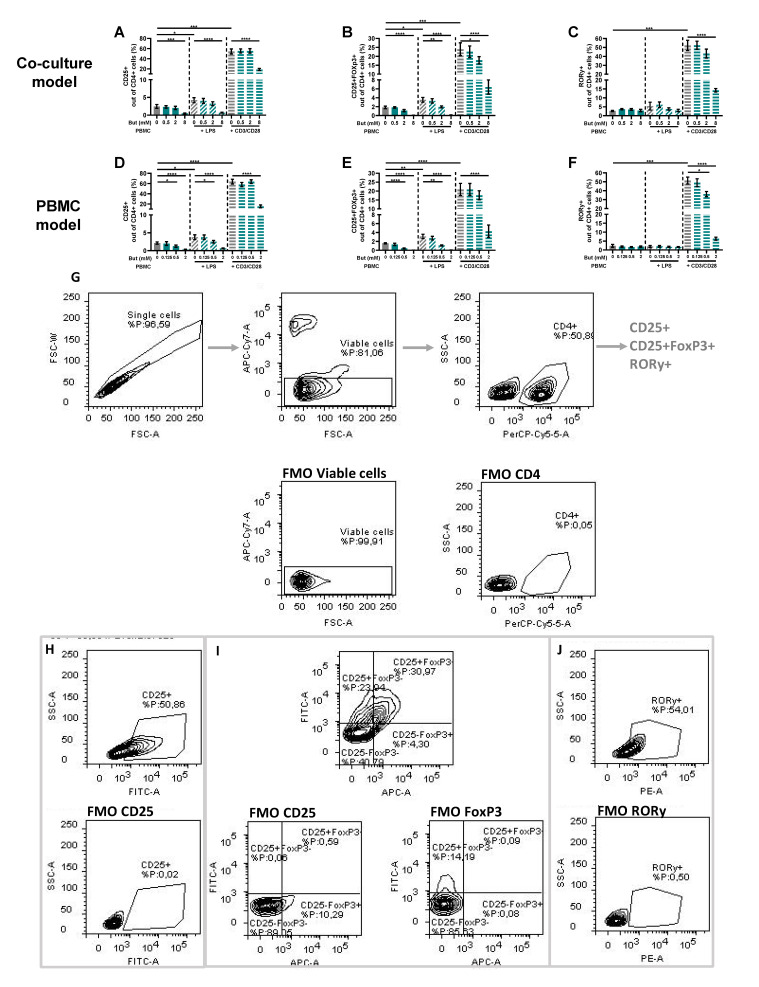
Butyrate modulates CD25+, CD25+FoxP3+ and RORγ+ cells in a Caco-2/PBMC co-culture model (**A**–**C**) and PBMC model (**D**–**F**). PBMCs were activated with LPS or αCD3/CD28 and cells were treated with 0–8 mM butyrate. (**G**) Used gating strategy of a representative sample with corresponding free minus one (FMO) sample, single cells gating > viable cells gating > CD4+ cells gating followed by CD25+ or CD25+FoxP3+ or RORγ+ gating. (**H**) CD25+ gating of a representative sample including FMO sample of CD25. (**I**) CD25+FoxP3+ gating of a representative sample including FMO sample of CD25 and FoxP3. (**J**) RORγ+ gating of a representative sample including FMO sample of RORγ. Data are presented as mean ± SEM and the datasets used for statistical analysis are divided by dotted lines (N = 6 individual experiments). Significant differences are shown as * *p* < 0.05, ** *p* < 0.01, *** *p* < 0.001, **** *p* < 0.0001 compared to control. But: butyrate; PBMC: peripheral blood mononuclear cells.

PBMCs were analyzed by means of flow cytometry to identify whether butyrate modulated the phenotype of the PBMCs. The LPS and αCD3/CD28-activated Caco-2/PBMC or PBMC model showed an increase in CD25+ activated T-cells and CD25+FoxP3+ regulatory T-cells, while only the αCD3/CD28 activated models showed an increase in RORγ+ T helper (Th)17-cells. Butyrate largely prevented these changes at concentrations of 2 and/or 8 mM. The percentages of CD25+ activated T-cells and CD25+FoxP3+ regulatory T-cells were even reduced by 2 mM butyrate in the control conditions using non-activated PBMCs (Figure 4).

**Figure 5:**
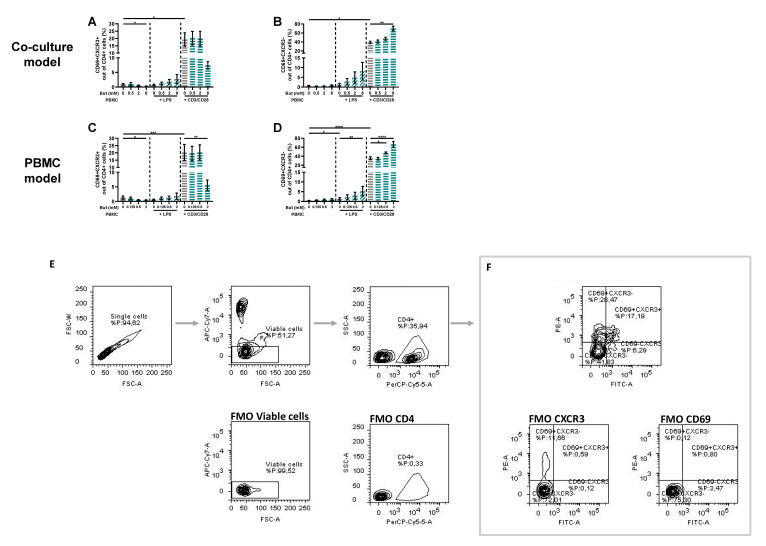
Butyrate modulates CD69+CXCR3+ and CD69+CXCR3− cells in a Caco-2/PBMC co-culture model (**A**,**B**) and PBMC model (**C**,**D**). PBMCs were activated with LPS or αCD3/CD28 and cells were treated with 0–8 mM butyrate. (**E**) Used gating strategy of a representative sample with corresponding free minus one (FMO) sample, single cells gating > viable cells gating > CD4+ cells gating followed by (**F**) CD69+CXCR3+ and CD69+CXCR3− gating. Data are presented as mean ± SEM and the datasets used for statistical analysis are divided by dotted lines (N = 6 individual experiments). Significant differences are shown as * *p* < 0.05, ** *p* < 0.01, *** *p* < 0.001, **** *p* < 0.0001 compared to control. But: butyrate; PBMC: peripheral blood mononuclear cells.

Th1-cells were analyzed in two panels, using CXCR3 or Tbet as Th1-type marker. In the panel with CXCR3 as Th-1 type marker, the αCD3/CD28-activated models showed an increase in both CD69+ activated CXCR3+ and CXCR3− Th-cells. Butyrate (8 mM) decreased the percentage of CD69+CXCR3+ Th1-cells, while increasing the percentage of CD69+CXCR3− Th-cells (Figure 5). In addition, butyrate showed a slight increase in CD69+CXCR3− cells in the LPS-activated PBMC model and a slight decrease in the non-activated models (Figure 5).

**Figure A1:**
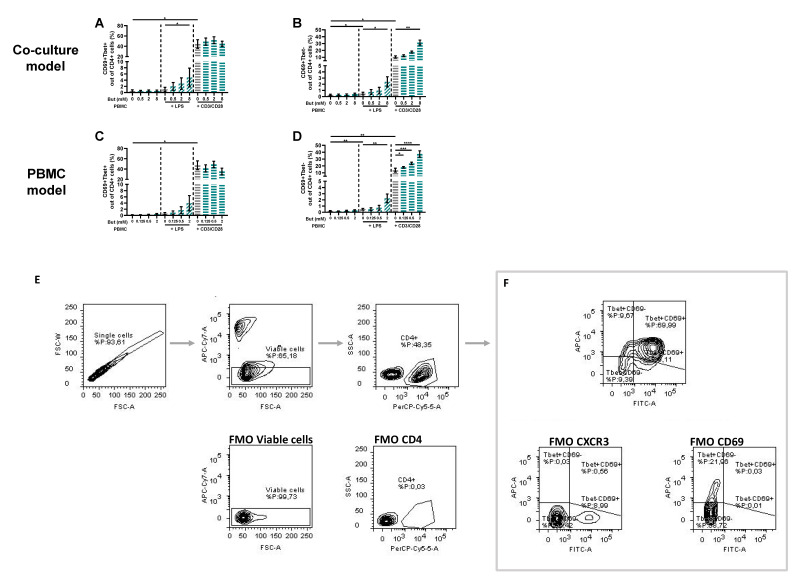
Butyrate modulates CD69+Tbet+ and CD69+Tbet− cells in a Caco-2/PBMC co-culture model (**A**,**B**) and PBMC model (**C**,**D**). PBMCs were activated with LPS or anti-CD3/CD28 and cells were treated with 0–8 mM butyrate. (**E**) Used gating strategy of a representative sample with corresponding free minus one (FMO) sample, single cells gating > viable cells gating > CD4+ cells gating followed by (**F**) CD69+Tbet+ and CD69+Tbet− gating. Data are presented as mean ± SEM and the datasets used for statistical analysis are divided by dotted lines (N = 6 individual experiments). Significant differences are shown as * *p* < 0.05, ** *p* < 0.01, *** *p* < 0.001, **** *p* < 0.001 compared to control. But: butyrate; PBMC: peripheral blood mononuclear cells.

In the panel with Tbet as Th1-type marker, the αCD3/CD28-activated models showed an increase in both CD69+ activated Tbet+ and Tbet− Th-cells as well. Contrary to the CXCR3+ panel, butyrate did not decrease the percentage of CD69+Tbet+ Th1-cells. Although, also here, the percentage of CD69+Tbet− Th-cells increased in the 8 mM butyrate conditions. In addition, butyrate showed a slight increase in CD69+Tbet+ and CD69+Tbet− cells in the LPS-activated models (Figure A1). 

### 3.4. SCFAs Reduce Epithelial Activation and Improve Barrier in a Caco-2 Monolayer

**Figure 6:**
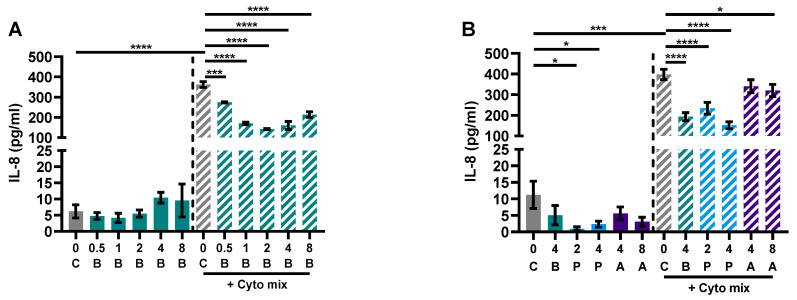
Butyrate (**A**,**B**), propionate and acetate (**B**) reduce IL-8 release of Caco-2 cells upon stimulation with pro-inflammatory cytokines. Data are represented as mean ± SEM of N = 4 individual experiments. * *p* < 0.05, *** *p* < 0.001, **** *p* < 0.0001 as compared to the control cells or cells exposed to the cyto mix (10 ng/mL TNF-α, 100 U/mL IFN-γ and 10 ng/mL IL-1β). The concentration of SCFAs is expressed in mM. A: acetate; B: butyrate; C: control; P: propionate.

The previous results using the co-culture model of IEC with activated PBMCs raised the question of whether the effect of butyrate on the barrier of the Caco-2 cells was an indirect effect via a decreased immune activation, less inflammation and thus reduced barrier disruption, or whether butyrate could protect against the barrier disruptive effect of the inflammatory mediators TNF-α, IFN-γ and IL-1β, which are known for their barrier disrupting capacities [42,43,44]. Caco-2 cells were incubated with different concentrations of butyrate, propionate and acetate. In addition to barrier function, epithelial activation was measured by means of IL-8 release in the basolateral compartment. Cells activated with the cytokine mix released a higher amount of IL-8 compared to controls and all concentrations (0.5–8 mM) of butyrate reduced IL-8 release; 2 and 4 mM propionate were equally able in reducing IL-8 release followed by 8 mM acetate; 4 mM acetate did not affect IL-8 release (Figure 6A,B).

**Figure 7:**
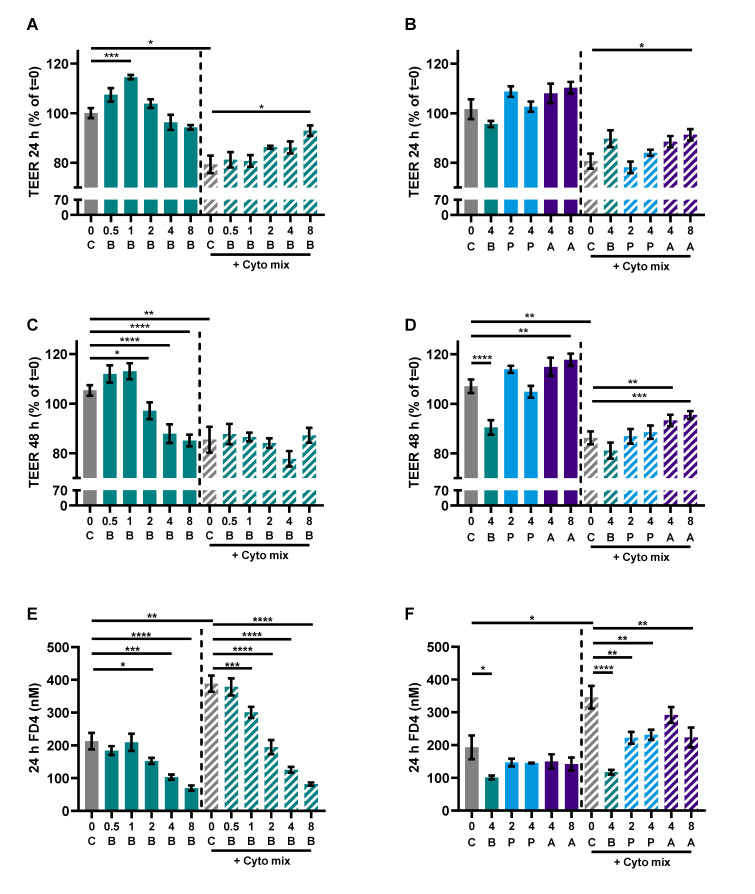
The effect of SCFAs on TEER measurements after 24 h (**A**,**B**) and 48 h (**C**,**D**) and FD4 permeability (**E**,**F**). Data are represented as mean ± SEM of N = 4 individual experiments. * *p* < 0.05, ** *p* < 0.01, *** *p* < 0.001, **** *p* < 0.0001 as compared to the control cells or cells exposed to the cyto mix (10 ng/mL TNF-α, 100 U/mL IFN-γ and 10 ng/mL IL-1β). The concentration of SCFAs is expressed in mM. A: acetate, B: butyrate, C: control, P: propionate.

**Figure A2:**
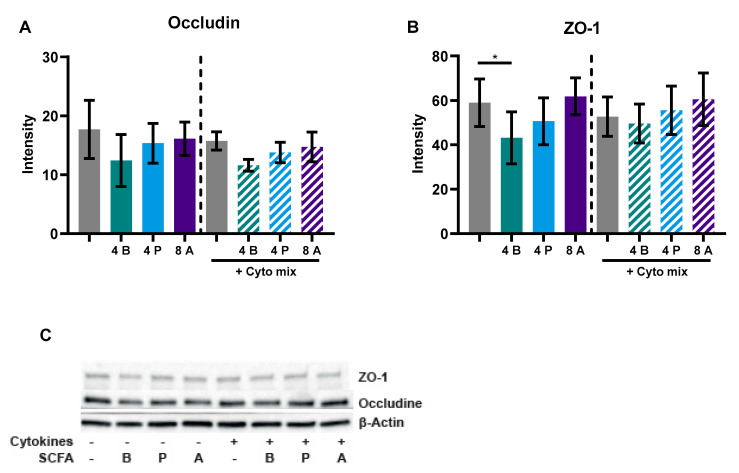
The effect of SCFAs on occludin (**A**) and ZO-1 (**B**) protein levels, including a representative western blot image (**C**). Data are represented as mean ± SEM of N = 4 individual experiments. Significant difference is shown as * *p* < 0.05 compared to control. Cells were treated with 4 mM butyrate, 4 mM propionate or 8 mM acetate and activated with a cytokine mix (10 ng/mL TNF-α, 100 U/mL IFN-γ and 10 ng/mL IL-1β). A: acetate; B: butyrate; P: propionate.

**Figure A3:**
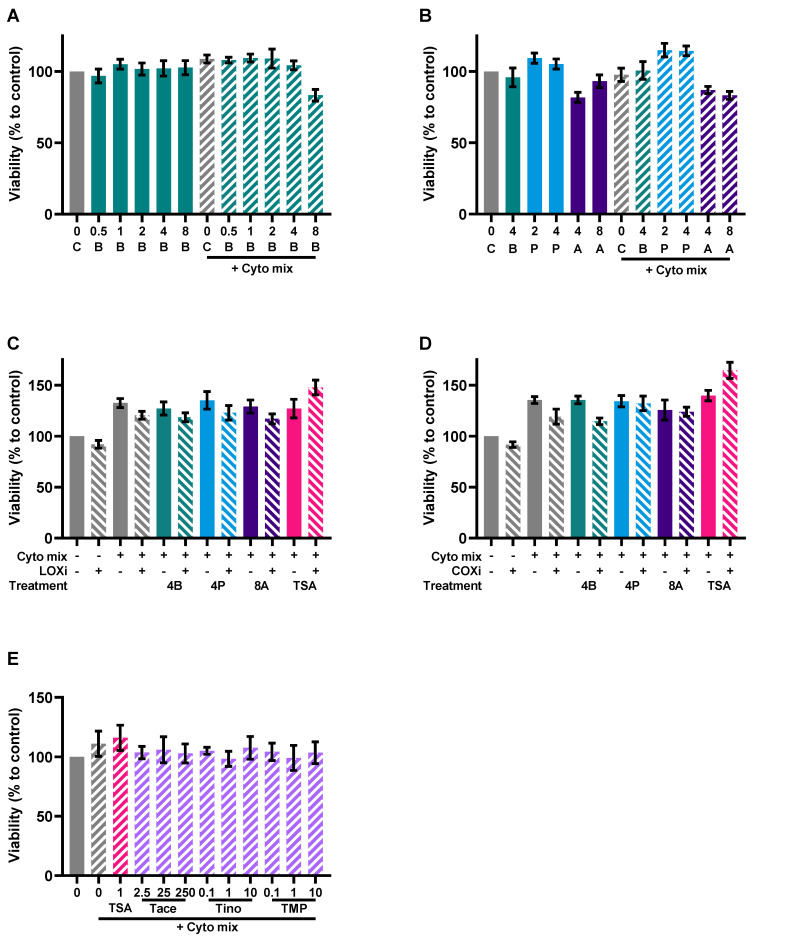
Viability of the Caco-2 cells after the experiments with butyrate (**A**), SCFA (**B**), a LOX inhibitor (**C**), a COX inhibitor (**D**) and the different HDAC inhibitors (**E**). Data are represented as mean ± SEM of N = 4 individual experiments. The concentration of SCFAs is expressed in mM, the concentrations of HDAC inhibitors in μM. A: acetate; B: butyrate; C: control; P: propionate; COXi: COX inhibitor indometacine; LOXi: LOX inhibitor nordihydroguaiaretic acid; Tace: tacedinaline; Tino: tinostamustine; TMP: TMP269; TSA: trichostatin A.

**Figure A4:**
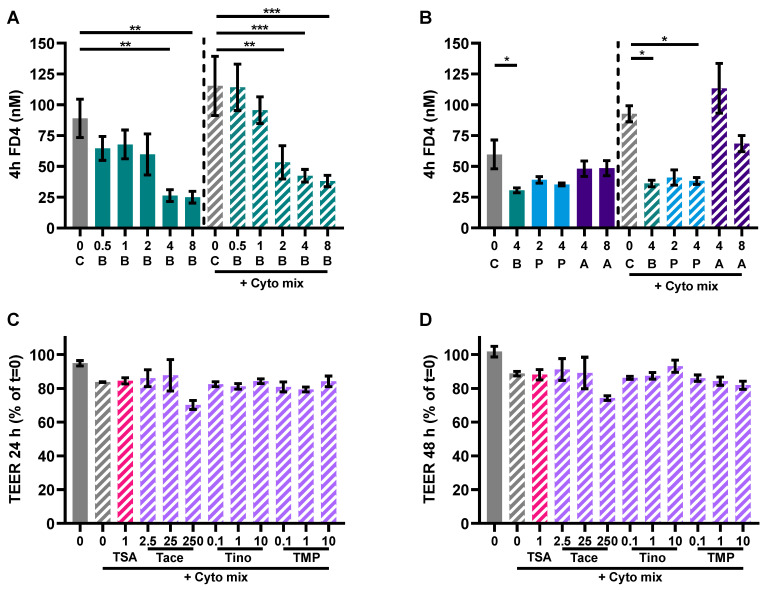
Additional barrier function measurements. (**A**,**B**) FD4 permeability sampled after 4 h in the experiment with Caco-2 cells activated with a cytokine mix (10 ng/mL TNF-α, 100 U/mL IFN-γ and 10 ng/mL IL-1β) and treated with butyrate, propionate or acetate. Data are represented as mean ± SEM of N = 4 individual experiments. * *p* < 0.05, ** *p* < 0.01, *** *p* < 0.001 as compared to the control cells or cells exposed to the cyto mix. (**C**,**D**) TEER values after 24 h and 48 h measured in Caco-2 cells activated with a cytokine mix and treated with different HDAC inhibitors. Data are represented as mean ± SEM of N = 3 individual experiments. * *p* < 0.05, ** *p* < 0.01, *** *p* < 0.001 as compared to the cells exposed to the cyto mix. The concentration of SCFAs is expressed in mM, the concentrations of HDAC inhibitors in μM. A: acetate; B: butyrate; C: control; P: propionate; COXi: COX inhibitor indometacine; LOXi: LOX inhibitor nordihydroguaiaretic acid; Tace: tacedinaline; Tino: tinostamustine; TMP: TMP269; TSA: trichostatin A.

Caco-2 cells activated with the cytokine mix resulted in decreased TEER at 24 and 48 h and an increased FD4 permeability. Butyrate only improved TEER in the activated cells at 24 and not at 48 h, but showed a dose-dependent reduction of FD4 permeability in the non-activated and activated cells. Propionate did not improve TEER at both timepoints, but reduced FD4 permeability in activated cells as well. Acetate increased TEER of activated and non-activated cells and reduced FD4 permeability of activated cells. Butyrate reduced FD4 permeability with more than 60%, while acetate only improved FD4 permeability by approximately 30%. FD4 permeability is a direct measure of how leaky the intestinal barrier is for antigen leakage from the gut lumen, while TEER is an indirect measure since it measures ion flux across tight junctions. FD4 permeability was also sampled at the earlier timepoint of 4 h (Figure A4A,B); these results show similar trends to the results of 24 h (Figure 7E,F). The effect of the SCFAs on the barrier cannot be explained by an increased expression in tight junction proteins occludin and ZO-1 (Figure A2). The WST assay showed that the treatments did not affect the viability of the cells (Figure A3A,B).

### 3.5. HDAC Inhibitors Mimic the Protective Effects of Butyrate on IL-8 Release and FD4 Permeability

**Figure 8:**
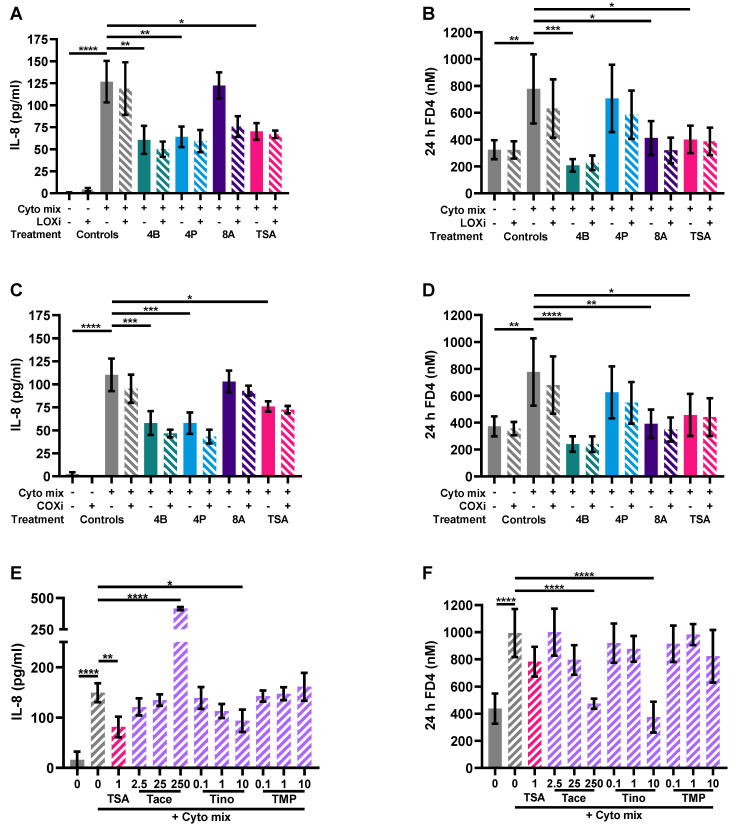
The effect of a lipoxygenase (LOX) inhibitor, cyclooxygenase (COX) inhibitor and histone deacetylase (HDAC) inhibitors on epithelial activation (**A**,**C**,**E**) and FD4 permeability (**B**,**D**,**F**). Data are represented as mean ± SEM of N = 4 individual experiments. * *p* < 0.05, ** *p* < 0.01, *** *p* < 0.001, **** *p* < 0.0001 as compared to cells exposed to the cyto mix (10 ng/mL TNF-α, 100 U/mL IFN-γ and 10 ng/mL IL-1β). The concentration of SCFAs is expressed in mM and of HDAC inhibitors in μM. A: acetate; B: butyrate; COXi: COX inhibitor indometacine; LOXi: LOX inhibitor nordihydroguaiaretic acid; P: propionate; Tace: tacedinaline; Tino: tinostamustine; TMP: TMP269; TSA: trichostatin A.

LOX, COX or HDAC inhibition might be involved in the mechanism of action of butyrate [26]. Therefore, we investigated whether this also applied in our model by adding a LOX inhibitor or COX inhibitor to butyrate-exposed cells or an HDAC inhibitor to mimic effects of butyrate which is known for its HDAC inhibitory capacities. Again, the cytokine mixture induced IL-8 release and enhanced FD4 permeability in the Caco-2 monolayer which were both largely prevented by 4 mM butyrate; 4 mM propionate lowered cytokine- induced IL-8 release similar to butyrate and 8 mM acetate protected against cytokine-induced increase in epithelial permeability. The LOX inhibitor nor COX inhibitor could counteract the protective effect of the SCFAs. However, HDAC inhibitor TSA mimicked the effect of butyrate by lowering the cytokine-induced IL-8 release and FD4 permeability (Figure 8A–F). Tinostamustine, a more selective HDAC inhibitor, which inhibits HDACs from class I and IIb showed a similar pattern. HDAC inhibitor TMP269, which inhibits HDACs from class IIa, was not effective. Tacedinaline, which inhibits HDACs from class I, decreased the cytokine-induced rise in FD4 permeability, but in contrast to butyrate, it increased IL-8 release at this concentration (Figure 8E,F). The different treatments did not affect viability of the cells (Figure A3C–E) and TEER was also not affected by the different treatments (Figure A4C,D).

## 4. Discussion

The intestinal barrier is one of the main defense mechanisms in the human body. It is getting more and more attention because it is shown that an impaired intestinal barrier is a common feature in non-communicable diseases (NCDs) [45,46,47,48]. The intestinal barrier consists of different layers including a mucosal layer, an epithelial cell monolayer and the lamina propria. The lamina propria lies beneath the epithelium and contains various effector immune cells, such as T-cells, B-cells, dendritic cells and macrophages [43]. The mucosal tissue plays a critical role in maintaining intestinal homeostasis. Intestinal epithelial cells prevent non-specific leakage of immunogenic agents such as endotoxin LPS by providing a barrier. In addition, the epithelial cells may regulate responses of the underlying immune cells. Immune cell activation in the lamina propria, however, can affect intestinal epithelial cell homeostasis since several cytokines, such as TNF-α, IL-1β, IFN-γ and IL-17a can activate epithelial cells and/or affect their barrier function [49]. Short chain fatty acids (SCFAs), in particular butyrate, have been shown to be able to contribute to intestinal homeostasis because they are known for their anti-inflammatory and intestinal barrier-supporting properties [24]. These effects were observed in in vitro models using intestinal epithelial cells or using immune cells alone, but it is unknown whether butyrate’s effect is strong enough to protect against immune-mediated barrier disruption in a co-culture model of intestinal epithelial cells (IEC) and activated immune cells (PBMCs). In the present study, PBMCs were activated by LPS or αCD3/CD28. LPS is recognized by immune cells through the pattern recognition receptor Toll-like receptor 4 (TLR4) expressed on the surface of mainly innate immune cells such as monocytes, macrophages and dendritic cells of which only monocytes are present in the PBMC mixture. Binding of LPS to TLR4 triggers signaling cascades that lead to the activation of transcription factors like nuclear factor kappa B (NF-κB) and the production of pro-inflammatory cytokines such as IL-1β, TNF-α, IL-6 and regulatory cytokine IL-10 [50]. 

αCD3/CD28 are monoclonal antibodies that bind to a surface receptor on T-cells. Anti-CD3 binds to the T cell receptor complex, while anti-CD28 binds to a co-stimulatory molecule. This combined activation leads to activation and the production of various cytokines such as TNF-α, IFN-γ, IL-17a and regulatory IL-10 by T-cells [51]. In the co-culture model, butyrate dose-dependently improved the intestinal barrier when the Caco-2 cell monolayer was disrupted by cytokine release from PBMCs activated with LPS or αCD3/28. Butyrate protected against decrease in TEER and suppressed cytokine responses in both situations, indicating the generic anti-inflammatory potency of butyrate on immune cells activated via different pathways. The barrier-protective effect of butyrate was observed before in various monoculture experiments using Caco-2 or T-84, while investigating its effect on the basal barrier or with different methods to disrupt barriers such as ethanol or inflammatory cytokines [27,28,29,52,53,54,55,56,57,58]. Only three other studies using co-culture models investigated the barrier-protective effect of butyrate. However, to our knowledge, we are the first to show barrier-protective effects at lower concentrations of butyrate (2 and 8 mM) in immune-mediated barrier disruption. The first study showed no statistically significant effect of butyrate in a model with Caco-2 and LPS-activated macrophages. However, the barrier was only assessed after 6 h [59], while in the present study, we showed the protective effect of butyrate after 24 and 48 h. The second study showed butyrate to improve the barrier in a model with Caco-2 and non-activated macrophage-like cells, but did not investigate the effect of butyrate in immune-mediated barrier disruption [60]. The third study showed only barrier-protective effects of high doses of butyrate (20 mM) in a model of Caco-2 cells with LPS-stimulated whole blood samples [61]. The current study focused on butyrate as it was observed before to be most beneficial in maintaining barrier properties during inflammation as compared to propionate and acetate [56,62,63]. For future studies, it would be interesting to compare the efficacy of butyrate, propionate and acetate alone as well as using these SCFAs combined in specific ratios known to be present in the intestinal lumen. This should be studied in in vitro models for inflammatory-induced barrier disruption combining IEC and immune cells, including the current LPS- or αCD3/28-activated PBMC/Caco-2 co-culture model or alternatively, instead of PBMCs, lamina propria-derived mononuclear cells may be used [64]. In addition, effects of SCFAs could be further studied in a co-culture model with Caco-2 and LPS-activated macrophages to further mimic the in vivo situation in the gut.

Although butyrate reduced barrier function as determined with TEER measurements in the Caco-2/non-activated PBMC model, it decreased functional permeability as indicated by a reduction in FD4 permeation into the basolateral compartment. TEER is an indirect indicator of barrier function since it measures ion fluxes over the tight junctions, and this can be disturbed, for example, by chloride secretion. FD4 is an inert direct marker of tight junction permeability and is used to confirm the TEER measurements. In this case, it shows that butyrate already affects basic barrier properties in absence of any inflammatory insult. This may also underly the protective effect of butyrate on barrier function when Caco-2 were exposed to activated PBMCs. However, beyond acting directly on the epithelial barrier, butyrate was also capable of lowering immune activation. 

PBMCs activated with LPS induced the release of IL-1β, IL-6, TNF-α and IL-10, whereas butyrate could only inhibit the release of TNF-α and IL-10. IL-1β and TNF-α are known to disrupt barriers [42,43,44], while IL-10 is known to promote barrier function [54]. In addition, TNF-α is found to synergize with other pro-inflammatory cytokines, such as IL-1β and IFN-γ, which could further enforce barrier disruption [65,66]. Butyrate-induced reduction in TNF-α by activated PBMCs may therefore be essential for its barrier-protective effects. Typically though, at the highest dose of butyrate, IL-1β release increased, while its barrier-protective effects were maintained. IL-10 is known for its barrier-protective effects, however, butyrate also lowered IL-10 secretion thus this could not explain the protective effect. Future studies should therefore focus on the underlying mechanism involved in butyrate’s barrier-protective effect in the higher dose range.

When compared to LPS, in addition to IL-6, TNF-α and IL-10, αCD3/CD28-activated PBMCs also induced IFN-γ and IL-17a. This is coherent as LPS mainly activates monocytes and αCD3/CD28 activates T-cells, which are capable of producing IFN-γ and IL-17a. Butyrate also inhibited the release of TNF-α and IL-10 by αCD3/CD28-activated cells similar to LPS-activated cells. In addition, butyrate inhibited IFN-γ and IL-17a release in the PBMC model, while in the Caco-2/PBMC co-culture, a similar declining pattern was observed. Therefore, like in the LPS-activated conditions, also here, the butyrate-induced suppression of TNF-α secretion by the activated immune cells may have largely contributed to the barrier-protective effects of butyrate.

The PBMC mixture contains monocytes, which is in contrast to the lamina propria which contains macrophages. The presence of inflammatory-type macrophages (M1) in the lamina propria can disturb immune homeostasis. Monocytes derived from the bone marrow can differentiate into macrophages in the intestinal tissue [67] and a reduction of TNF-α producing monocytes may suggest that butyrate may also control M1-type activation hereby contributing to intestinal homeostasis. This is in line with previous research showing that butyrate inhibits NF-κβ activation in macrophages in the lamina propria of patients with ulcerative colitis [68]. Furthermore, macrophages exposed to butyrate showed induced antimicrobial activity, contributing to maintain intestinal homeostasis [69].

In the αCD3/CD28-activated PBMCs, butyrate reduced Th1- and Th17-type cytokines IFN-γ and IL-17A secretion. Indeed, butyrate lowered the frequency of activated Th1 cells as shown by the percentage of CD4+CD69+CXCR3+ cells, although the percentage of CD4+CD69+Tbet+ cells remained unaltered. The reduction in IL-17a, however, was associated with the butyrate-induced reduction in the percentage of CD4+RORγ+ cells, which links to reduced Th17 activation.

The inhibitory effect of butyrate on Th1-cells, Th17-cells and pro-inflammatory cytokines secretion was observed previously, but the reduction in regulatory T-cells and IL-10 was different from most other publications [34,35,36,37,39]. However, depending on the dose, differential immune effects of butyrate have been shown previously. For example, IL-10 release was induced by 0.25 mM butyrate in αCD3-activated PBMCs, while IL-10 release was inhibited by 1 mM butyrate [36]. Contrary, 1–2 mM butyrate induced the release of IL-10 and the percentage of regulatory T-cells in LPS-activated PBMCs (5 μg/mL), while 0.2–20 mM butyrate inhibited IL-10 release in LPS-activated monocytes and PBMCs (0.5 μg/mL), showing no statistically significant effect on non-activated cells [35,37]. This suggests that the effect of butyrate not only is dependent on the concentration of butyrate present, but also on the type and strength of immune activation.

Typically though, butyrate increased expression of activation marker CD69+ on CD4 cells, which could not be linked to either Foxp3+ natural regulatory T-cells, Th1 nor Th17 cells. On the other hand, the expression of CD25 was reduced. Beyond FoxP3+ regulatory T-cells, other types of regulatory T-cells exist. In this respect, CD4+CD25−CD69+ cells have been previously indicated as a novel type of regulatory T-cells and can suppress T-cell proliferation in a cell–cell contact manner, which may explain the reduced percentages of Th-cells and regulatory T-cells (CD4+CD25+FoxP3+) [70]. Although, not a lot is known about this novel type of regulatory T-cells therefore more research would be needed to identify if butyrate affects these types of cells and how they can contribute to intestinal immune homeostasis.

LPS and αCD3/CD28 activation are complementary since the immune cascade is initiated from either the innate or the adaptive immune compartment. LPS and αCD3/CD28 both showed to produce different inflammatory cytokine patterns which could be suppressed by butyrate. Low-grade inflammation can lead to leaky gut and LPS leakage. LPS may activate innate immune cells which further contributes to chronic low-grade inflammation increasing the risk of NCDs. In immune-mediated NCDs, T-cell activation also increases inflammatory cytokines that may also contribute to a leaky gut [45,46,47,48]. Therefore, both activation pathways used in our studies are relevant when considering immune activation in NCDs. The present study shows that butyrate suppressed LPS and αCD3/CD28 induced immune activation and protected against inflammatory-induced barrier disruption, which shows the broad anti-inflammatory potential of butyrate. Similar effects were observed in different murine models of NCDs. Mice treated with butyrate in drinking water showed improved skin and intestinal barriers, which contributed in reducing the development of diseases such as arthritis and atopic dermatitis [71,72]. In addition, dietary supplementation of butyrate to mice fed with a high-fat diet proved to act as an anti-inflammatory [73,74]. 

TNF-α, IL-1β and IFN-γ are known to disrupt the intestinal barrier and IL-10 can promote barrier function [42,43,44,54]. As discussed before, butyrate partially reduced the release of these cytokines by activated PBMCs, and therefore the effect of butyrate on the intestinal barrier in the co-culture model might be indirect via its anti-inflammatory properties. To further investigate the direct barrier-protective effect of butyrate, Caco-2 were exposed to a cytokine mixture of TNF-α, IFN-γ and IL-1β. In this model, butyrate again proved its barrier-protective effects and its anti-inflammatory capacity by inhibiting IL-8 release, similar to previous observations [27,28,31,53,75]. Compared to propionate and acetate, butyrate was most effective since it both improved barrier function under inflammatory conditions and suppressed epithelial cell activation which is important to maintain homeostasis.

Cytokine-mediated barrier disruption often results in reduced expression of tight junction proteins like ZO-1 and occludin [76]. In our study, the cytokine mixture did not decrease the level of these tight junction proteins and butyrate, propionate and acetate also did not affect these protein levels. It could be that the barrier-supportive effects of the SCFAs butyrate and acetate result from more dense tight junction reassembly [28] or that the expression of other tight junction proteins is affected. For example, it was shown that butyrate increased Claudin-1 expression in the cdx2-IEC cell line and that it induced redistribution of ZO-1 and Claudin-1 [75].

Butyrate is the most potent HDAC inhibitor, which suggests the potential involvement of HDAC in the mechanism of action of these SCFAs [24,30,77,78]. Another study described the potential involvement of LOX and COX in the mechanism of action of butyrate [26]. In the present study, LOX and COX were not involved in the anti-inflammatory effects or the mechanism of barrier protection by the SCFAs. Contrary, general HDAC inhibitor TSA showed similar effects on barrier improvement and reduction in inflammatory-induced IL-8 release as butyrate, indicating that HDAC inhibition might indeed be involved in the mechanism of action of the SCFAs. More specific HDAC inhibitors showed various effects. Class IIa (HDAC 4, 5, 7, 9) HDAC inhibitor TMP269 showed no statistically significant effect on barrier integrity and IL-8 release, while the highest concentration of class IIb (HDAC 1, 2, 3, 8) and I (HDAC 6, 10) HDAC inhibitor tinostamustine showed a protective effect on barrier integrity and, albeit not significant, a similar pattern in IL-8 release as compared to butyrate. Strikingly, HDAC inhibitor tacedinaline, specifically inhibiting HDAC 1, 2 and 3 also had barrier-protective effects similar to butyrate, but it increased IL-8. Butyrate, propionate, TSA and a class II HDAC inhibitor previously have been shown to lower IL-8 release in stimulated Caco-2 cells [79,80]. However, general HDAC inhibitor TSA was also found to increase IL-8 levels in some conditions [81]. To our knowledge, here it is shown for the first time that HDAC inhibition can protect against inflammatory-induced barrier disruption similar to butyrate. Butyrate is known as a potent inhibitor of HDAC 2, 3 and 8, followed by 1, 4 and 5 to a lesser extent. In comparison to the HDAC inhibitors used to mimic the effect of butyrate, inhibition of HDAC 1, 2, 3, 4 and 5 did not show similar effects to butyrate and are therefore most probably not involved in its barrier-protective effect. While in comparison to the HDAC inhibitors used, a HDAC inhibitor inhibiting HDAC 8 did show similar effects to butyrate. Our results suggest that the beneficial effect of butyrate on intestinal barrier function and epithelial activation is likely mediated by the inhibition of specific HDACs, of which HDAC 8 would be the most promising candidate. The effect of specific HDAC 8 inhibitors has recently been investigated in a murine model of colitis showing barrier-improving effects via upregulation of occludin [82]. To the best of our knowledge, specific HDAC inhibitors were not investigated in immune-mediated barrier disruption in vitro and the potential involvement of HDAC 8 was shown here for the first time. 

Butyrate concentrations in the small intestine range from 0 to 26 mM from beginning to end and are highly impacted by diet and bacterial fermentation [83,84]. A change in diet or bacterial composition could therefore potentially result in reduced butyrate concentrations. In the present study, butyrate was found to have anti-inflammatory and barrier-protective effects in the range of 4–8 mM. Our work shows the importance of sufficient butyrate levels. This can be achieved by consuming a diet rich in fermentable fibers or by butyrate supplementation via nutritional supplements or by a pharmaceutical drug product releasing sufficient amounts of butyrate.

## 5. Conclusions

These findings highlight that butyrate plays an important role in maintaining both barrier integrity as well as immune homeostasis. This emphasizes the importance of having sufficient intestinal butyrate levels. Here, the essential role for butyrate in controlling gut health was shown in an experimental Caco-2/PBMC co-culture model allowing for the cross talk between epithelial cells and activated immune cells. In addition, butyrate directly inhibited the inflammatory response of activated PBMCs. Butyrate proved not only to reduce barrier disruption via lowering local inflammatory responses, but also had a direct protective effect on cytokine-mediated barrier disruption. The effect of butyrate is most probably mediated via HDAC, of which HDAC 8 inhibition may be the main target in controlling both barrier as well as inflammation. In conclusion, the HDAC inhibitory effect of butyrate may protect against both inflammatory-induced barrier disruption as well as immune activation and can therefore have a protective role in NCDs.

## Data Availability

The data presented in this study are available upon request from the corresponding author.

## Abbreviations

COX	cyclooxygenase
CXCL10	C-X-C motif chemokine ligand 10
ELISA	enzyme-linked immunosorbent assay
FD4	4 kDa fluorescein isothiocyanate-dextran
FMO	free minus one
HDAC	histone deacetylase
IEC	intestinal epithelial cells
IFN-γ	interferon-gamma
IL	interleukin
LOX	lipoxygenase
NCDs	non-communicable diseases
NF-κB	nuclear factor kappa B
PBMCs	peripheral blood mononuclear cells
PBS	phosphate buffered saline
SCFAs	short chain fatty acids
TEER	transepithelial electrical resistance
Th	T helper
TLR4	toll-like receptor 4
TNF-α	tumor necrosis factor-alpha
ZO-1	zonula occludens-1
